# Potential Serological Misdiagnosis of Barmah Forest Virus and Ross River Virus Diseases as Chikungunya Virus Infections in Australia: Comparison of ELISA with Neutralization Assay Results

**DOI:** 10.3390/v16030384

**Published:** 2024-02-29

**Authors:** Joanne Kizu, Melissa Graham, Wenjun Liu

**Affiliations:** 1 Australian Defence Force Malaria and Infectious Disease Institute, Weary Dunlop Drive, Gallipoli Barracks, Enoggera, QLD 4051, Australia; joanne.kizu@defence.gov.au (J.K.); melissa.graham@defence.gov.au (M.G.); 2 Queensland Institute of Medical Research-Berghofer Medical Research Institute, Herston, Brisbane, QLD 4006, Australia

**Keywords:** antibody, Barmah Forest virus, Chikungunya virus, neutralizing antibody, Ross River virus, misdiagnosis

## Abstract

To evaluate the frequency of errors in the diagnosis of medical laboratory-diagnosed Chikungunya virus (CHIKV) infections in Australia, we studied 42 laboratory-diagnosed CHIKV serum samples from one Queensland medical laboratory by ELISA IgG/IgM and measured the specific neutralization antibodies (Nab) against Barmah Forest virus (BFV), CHIKV and Ross River virus (RRV). The sero-positivity rates for the sera were as follows: anti-BFV IgG^+^ 19% (8/42), IgM^+^ 2.4% (1/42) and Nab^+^ 16.7% (7/42); anti-CHIKV IgG^+^ 90.5% (38/42), IgM^+^ 21.4% (9/42) and Nab^+^ 90.5% (38/42); anti-RRV IgG^+^ 88.1% (37/42), IgM^+^ 28.6% (12/42) and Nab^+^ 83.2% (35/42), respectively. Among the samples with multiple antibody positivity, 2.4% (1/42) showed triple ELISA IgM^+^, and 14.3% (6/42) exhibited double IgM RRV^+^CHIKV^+^; 9.5% (4/42) showed triple IgG^+^, 76.2% (32/42) displayed double IgG RRV^+^CHIKV^+^, 4.8% (2/42) showed IgG BFV^+^RRV^+^ and 4.8% (2/42) showed IgG BFV^+^+CHIKV^+^; and 9.5% (4/42) showed triple Nab^+^ and 69% (29/42) exhibited double Nab RRV^+^CHIKV^+^, respectively. Our analysis of the single-virus infection control Nab results suggested no cross-neutralization between RRV and BFV, and only mild cross-neutralization between CHIKV and RRV, BFV and CHIKV, all with a ≥4-fold Nab titre ratio difference between the true virus infection and cross-reactivity counterpart virus. Subsequently, we re-diagnosed these 42 patients as 1 BFV^+^, 8 CHIKV^+^ and 23 RRV^+^ single-virus infections, along with five RRV^+^/BFV^+^ and four RRV^+^/CHIKV^+^ double infections, and one possible RRV^+^/BFV^+^ or RRV^+^CHIKV^+^, respectively. These findings suggests that a substantial proportion of medically attended RRV and BFV infections were misdiagnosed as CHIKV infections, highlighting the imperative need for diagnostic laboratory tests capable of distinguishing between CHIKV infections and actively co-circulating RRV and BFV. For a correct diagnosis, it is crucial to consider reliable diagnostic methods such as the neutralization assay to exclude RRV and BFV.

## 1. Introduction

Barmah Forest virus (BFV), Chikungunya virus (CHIKV) and Ross River virus (RRV) are closely phylogenetically and antigenically related mosquito-borne, single-stranded, positive-sense RNA viruses that belong to the *Alphavirus* genus of the family *Togaviridae* [1,2]. Phylogenetically, these three alphaviruses belong to one sero-complex Semliki virus clade of the *Alphavirus* genus. RRV and BFV, recognized as typical Australian arboviruses, are the top two causes of human arboviral diseases in Australia, with ~55,000 RRV and ~15,000 BFV cases reported over the past decade [3]. CHIKV has caused large epidemics linked to acute, chronic, and severe clinical outcomes in humans [4,5]. After major outbreaks starting in 2004, CHIKV has spread to subtropical areas and the western hemisphere from sub-Saharan Africa, Southeast Asia, and the Indian subcontinent, and has caused millions of human infections around the world. Although it is not endemic in Australia, cases imported from Pacific Ocean Island countries and Territories into Australia have been reported from time to time [6,7]. RRV and BFV have a natural animal–mosquito–animal transmission cycle throughout Australia [8,9], whereas CHIKV maintains a sylvatic cycle with nonhuman primates, which are thought to be the major reservoir host [10]; human–mosquito–human transmission is the major pathway for infection during epidemics. About 55–75% of RRV infections in humans are asymptomatic or subclinical infection. The number of asymptomatic BFV infections has not been formally reported but is likely to be similar or higher than that for RRV [11]. The number of asymptomatic cases for CHIKV infection is approximately 3–28% [12]. For people who develop symptomatic illness, the incubation period of these three viruses is similar, typically 3–7 days (range 1–13 days). These viruses cause similar symptoms that are often characterized by the sudden onset of high fever, maculopapular erythematous rash on the limbs and trunk, headache, fatigue, conjunctivitis, myalgia and joint pains, and are often clinically indistinguishable. Fevers typically last for ≤1 week, but the joint pain symptoms can be severe, usually involving multiple joints, typically bilateral and symmetric. There are no specific drugs or licensed vaccines available to prevent the diseases caused by these three alphaviruses. Clinical management primarily targets the relief of symptoms. Most of the infections are self-limiting; recovery is expected within 4–6 weeks after symptom onset. However, arthralgia and myalgia-associated persistent joint pain can last for over six months in about 10–20% of RRV/BFV cases and in 5–80% of CHIKV patients. Compared to RRV disease, BFV disease may have slightly milder symptoms, with less joint swelling and a shorter duration of chronic disease [13]. CHIKV disease might present more severe and debilitating symptoms that vary in duration. Co-circulation with other mosquito-borne viruses such as dengue virus can make clinical diagnosis even more challenging since these virus infections may cause similar clinical symptoms [14,15]. As the clinical symptoms of these three alphavirus infections are similar, differential diagnostic methods able to identify the true virus infection are necessary for better clinical management, public alert and targeted vector control in epidemic areas to prevent further spread. Infection with these viruses likely confers life-long immunity, in which neutralizing antibodies prevent disease recurrence, as re-infection has not been reported [16,17]. These alphavirus infections are notifiable diseases in Australia.

In accordance with the Australian Health Department, the definitive laboratory diagnosis of CHIKV infection requires evidence of virus isolation, or the detection of viral RNA by RT-PCR testing in serum collected <6 days after the onset of illness. Diagnosis by serological methods requires seroconversion or a ≥4-fold rise in IgG titres in the absence of a corresponding change in antibody levels to RRV and BFV, or the detection of CHIKV IgM in the absence of anti-RRV and anti-BFV IgM [18]. CHIKV virus isolation must be handled under biosafety level (BSL) three laboratory restrictions in Australia. The detection of particular RRV/BFV-specific IgM and IgG antibodies in a single serum would be only suggestive of infection according to the Australian Department of Health [19], possibly due to cross-reactivity-related false positive or false negative results among these alphaviruses and possible flaviviruses [20]. We recommend that this definition should be applied to CHIKV disease as well. As alphavirus viremia is short-lived, neither virus isolation nor real-time reverse transcription polymerase chain reaction (RT-PCR) assays are currently performed in Australian public laboratories. Clinicians in Australia largely depend on the pathology laboratory detection of CHIKV-specific IgM/IgG antibodies using commercially available kits, which normally appear in serum collected approximately >5 days after illness onset [21,22]. Due to the broad IgM/IgG cross-reactivity among these alphaviruses and flaviviruses and the laboratory diagnosis of three virus infections predominantly by ELISA IgM/IgG-based testing, there will inevitably be ambiguity in disease recognition in Australia for these alphaviruses.

We assessed the frequency of errors in the diagnosis of 42 CHIKV^+^ serum samples from a Queensland medical laboratory, originally diagnosed by their in-house-made IgG/IgM EIA in comparison with Euroimmun’s ELISA IgM/IgG assays. Our assessment was a cross-sectional study conducted by using ELISA IgG/IgM and measuring specific neutralization antibodies (Nab) against all three alphaviruses.

## 2. Methods

### 2.1. Ethical Approval

The study was approved by the Department of Defence and Veteran Affairs Human Research Ethics Committee (DDVA HREC 153-19, and HREC 157-19). The patients consented to the use of their serum to validate the laboratory serological diagnosis methods.

### 2.2. Patient Samples

We obtained 42 patient CHIKV serum samples collected by one Queensland medical laboratory from 9 July 2020 to 24 July 2021. Our initial purpose was to use these validated samples to check the cross-neutralization among these three alphaviruses.

### 2.3. Positive and Negative Control Samples

Fourteen RRV and six BFV single-virus-infected Australian Defence Force (ADF) soldier serum samples were used as positive controls. These serum samples were identified as a single-virus infection by either RT-PCR or virus isolation, or as a previous ≥4-fold rise in Nab titres against either RRV or BFV in our laboratory. Two CHIKV single-infected serum samples were obtained from a Queensland public pathology laboratory. Both sera were diagnosed as CHIKV infections using the Euroimmun ELISA IgM kit, and this was further confirmed by a neutralization assay against BFV, CHIKV and RRV. The Panbio RRV IgG kit positive control was also included. Five ELISA IgG/IgM negative to all three alphaviruses ADF sera were used as negative controls.

### 2.4. Cells and Viruses

Vero cells (African green monkey kidney epithelial cells) were purchased from the American Type Culture Collection (ATCC, CCL-81) and cultured in 10% *v*/*v* heat-inactivated foetal calf serum (FCS, Life Technologies, Carlsbad, CA, USA)/RPMI 1640 medium (Sigma-Aldrich, St. Louis, MO, USA). An RRV QML strain (GenBank No. GQ433354), a prototype BFV strain (BH2193, GenBank No. NC_001786) and a CHIKV Reunion strain (GenBank No. DQ443544) were used for the neutralizing antibody (Nab) assays. The virus strains were passaged three times in Vero cells and the cell culture supernatant was harvested, aliquoted, and stored at −80 °C for further use. One vial of viral stock was thawed to determine the virus titre using a 50% tissue culture infectious dose (TCID50/mL) on Vero cells, as described in our previous publication [23]. The TCID50/mL was calculated according to the published Reed–Muench method [24].

### 2.5. ELISA

Commercially available semi-quantitative ELISA anti-BFV IgG/IgM (product code IgG/SD05PE10, IgM/05PE20) and anti-RRV IgG/IgM (product code IgG/04PE10, IgM/04PE20) kits from Abbott, Australia (Abbott, https://www.globalpointofcare.abbott/en/index.html, accessed on 24 January 2024), and anti-CHIKV ELISA IgG/IgM kits (product code EI 293A-9601G and EI 293A-9601M) from Euroimmun (Euroimmun, https://www.euroimmun.com, Lübeck, Germany, accessed on 24 January 2024) were used according to the manufacturer’s protocol. Positivity was determined by comparing the sample result to the IgG/IgM reference sera provided (cut-off calibrators). A positive sample was defined as having a sample/calibrator absorbance ratio of ≥1.1; a negative sample was defined as having a ratio of <0.8; and an equivalent sample was defined as having a ratio of ≥0.8 to <1.1.

### 2.6. Micro-Neutralization Assay

The anti-BFV, anti-CHIKV and anti-RRV-specific Nab responses were assessed using a micro-neutralization assay, as described in our previous publication [23]. Briefly, heat-inactivated (56 °C for 30 min) serum samples were diluted 1:10 with dilution medium (DM) (RPMI supplemented with 10% foetal bovine serum, 10 mM of HEPES, 20 mM of L-glutamine and 100 units/mL of penicillin). Two-fold serial dilutions were performed in duplicate using 50 μL aliquots across rows A–G of the plate. Wells G1–G6 of the plate contained 50 μL aliquots of DM alone as virus controls, and wells G7–G12 contained 100 μL aliquots of DM as cell-only controls.

Volumes of 50 μL of virus stock, containing 100 TCID50 infectious doses of either BFV, CHIKV or RRV, were added to the virus control wells and to those with serially diluted sera, but not to the cell-only control wells. The mixtures were incubated at 37 °C with 5% CO_2_ for 1 h to allow virus neutralization. Following neutralization, a suspension of 2 × 10^4^ Vero cells in 100 μL of DM was added into each well of the 96-well plates. Ninety-six hours later, the cells were fixed with 3.7% formaldehyde, then stained with 1% crystal violet for 1 h, washed in tap water and dried; then, the absorbance (O.D.) of the wells was read at 595 nm using a 96-well plate spectrophotometer (Infinite M200 pro, TECAN, Männedorf, Switzerland). The test results were considered valid if the O.D. of the virus control wells in the same plate was lower than 0.3 and if the O.D. of the cell control wells was higher than 1.5. The value used to determine the presence of neutralizing antibodies was calculated according to the following formula: cut-off value = (mean O.D. of 4 virus control wells in the same plate + mean O.D. of 4 cell control wells in the same plate)/2. If the duplicate wells were both above the cut-off values, the reciprocal antibody dilution in those wells was recognized as a neutralization antibody titre for that human sample. Anti-BFV-, anti-CHIKV- or anti-RRV-specific Nab titres ≥ 10 were considered positive.

### 2.7. Single Virus Infection and Multiple Virus Infections

Single and multiple virus infections were defined as described in our previous publication [23]. Briefly, this is Nab titres ≥ 10 to only one alphavirus or titres ≥ 10 to multiple viruses but with a predominant response (≥4 times the difference between RRV and CHIKV, CHIKV and BFV Nab titres, respectively).

BFV^+^RRV^+^ double infection was defined as both RRV^+^BFV^+^-positive samples, or RRV^+^BFV^+^CHIKV^+^ samples with the Nab titre of RRV^+^/CHIKV^+^ ≥4-fold or BFV^+^/CHIKV^+^ ≥4-fold.

RRV^+^CHIKV^+^ double infection was defined as RRV^+^CHIKV^+^ samples without a single predominant Nab titre, or CHIKV/BFV ≥4-fold plus RRV/CHIKV ≤4-fold in triple Nab^+^ samples.

## 3. Results

### 3.1. Study Population Demographics

We collected 42 patient serum samples that had initially been diagnosed as CHIKV infections using an in-house IgM/IgG EIA and Euroimmun ELISA IgG/IgM methods between 9 July 2020 and 24 July 2021. However, the detailed original laboratory serological results were not available. These patients ranged in age from 12 to 81 years, with a mean age of 46.5, and comprised 29 male and 13 females. Among them, 28 patients had clinical descriptions, with the most frequent symptoms being polyarthritis stiffness in eight cases, fever and rash in five cases, muscle pain in five cases, and fatigue and headache in six cases. Clinical data were available for fourteen patients upon request. Notably, three patients had a clear travel history to Indonesia, India, or Sri Lanka and had returned to Australia exhibiting symptoms of joint pain and poly-arthralgia.

### 3.2. ELISA IgG/IgM and Pathogen-Specific Nab of Postive and Negative Control Results

Fourteen single RRV virus-infected positive controls (14 IgG^+^ and 10 IgM^+^) showed six cases of anti-CHIKV IgG^+^ and six cases of IgM^+^; among them, eight showed the cross-neutralization of CHIKV Nab, all with low Nab titres ≤20 and an RRV/CHIKV Nab titre ratio of ≥4-fold. Among these RRV controls, one showed anti-BFV IgG^+^ and one IgM^+^, yet none of these 14 RRV-positive control samples, nor Panbio-positive control samples, exhibited cross-neutralized BFV.

Six single BFV-infected controls (6 IgG^+^ and 2 IgM^+^) showed anti-RRV activity, with two cases of IgM^+^ and IgG^±^; however, none of these samples neutralized RRV. Notably, one sample among these single BFV virus infection controls cross-neutralized CHIKV, with a low Nab titre of 10, along with a titre ratio of BFV/CHIKV of ≥4-fold.

Two single CHIKV-infected controls (both IgG^+^IgM^−^) showed both anti-RRV IgG^+^IgM^−^ and cross-neutralized RRV, with Nab titres of ≤20 and an CHIKV/RRV Nab titre ratio of ≥4-fold, but these did not neutralize the BFV prototype strain.

Five ADF sera controls collected before being deployed to PNG in 2019 tested negative on ELISA and did not neutralize any virus (Table 1).

### 3.3. Pathogen-Specific ELISA IgG/IgM and Pathogen-Specific Nab Results of 42 CHIKV Samples

The ELISA IgG/IgM and Nab antibody characteristics, along with the potential true virus exposures of the patients, are presented in Table 2. In summary, the prevalence of anti-BFV antibodies is as follows: IgG^+^ 19% (8/42), IgM^+^ 2.4% (1/42) and Nab^+^ 16.7% (7/42) (with a mean 160, 95% CI: -43.5–363.5), respectively. One sample exhibits both anti-BFV IgG^+^ and IgM^+^, while one sample tests positive on ELISA but negative on Nab (Table 3).

Regarding anti-CHIKV antibodies, the prevalence rates are as follows: IgG^+^ 90.5% (38/42), IgM^+^ 21.4% (9/42) and Nab^+^ 92.6% (39/42) (with a mean titre 85.1, 95% CI: 36.4–133.8), respectively. Nine samples show positivity for both anti-CHIKV IgG^+^ and IgM^+^. Additionally, two samples test positive on ELISA but negative on Nab, while two are negative on ELISA but positive on Nab (Table 3).

For anti-RRV antibodies, the prevalence rates are as follows: IgG^+^ 88.1% (37/42), IgM^+^ 28.6% (12/42) and Nab^+^ 83.3% (35/42) (with a mean titre: 251.1, 95% CI: 186.7–315.8), respectively. Twelve samples exhibit positivity for both anti-RRV IgG^+^ and IgM^+^, while two are positive on ELISA but negative on Nab (Table 3).

The statistical analysis revealed no significant difference in a comparison of RRV sero-positivity (IgG/IgM and Nab) with that of CHIKV (chi square tests). However, the anti-RRV Nab titre is significantly higher than that of anti-CHIKV (Two-tailed *t* test, *p* = 0.0007, *p* ≤ 0.5).

Table 4 summarizes the occurrence of multiple antibody positivity observed in a significant number of samples. For IgG, these numbers are as follows: BFV^+^/CHIKV^+^ in 4.8% (2/42) of samples, BFV^+^/RRV^+^ in 2.4% (1/42), CHIKV^+^RRV^+^ in 71.4% (30/42), and BFV^+^CHIKV^+^RRV^+^ in 11.9% (5/42) of samples. For IgM, the occurrences are CHIKV^+^RRV^+^ in 11.9% (5/42) of samples and BFV^+^/CHIKV^+^/RRV^+^ in 2.4% (1/42) of samples. In terms of Nab, the occurrences are CHIKV^+^/RRV^+^ in 69.1% (29/42) of samples and BFV^+^/CHIKV^+^/RRV^+^ in 11.9% (5/42) of samples (Table 4).

### 3.4. The Possible Diagnosis of These Samples

Based on the differences in the Nab titres of our single-virus-infected positive control samples, we re-evaluated these 42 cases initially diagnosed as CHIKV according to the definitions for single and multiple infections outlined in our previous publication [23] and detailed in the Materials and Methods section. Sample No.23 exhibited a single BFV Nab^+^, suggesting a potential BFV infection. Among the other serum samples, six samples displayed single anti-CHIKV Nab^+^, while two (No. 13 and No. 39) showed anti-CHIKV^+^RRV^+^, with the CHIKV/RRV Nab titre > 4. Consequently, these eight samples were classified as potential single CHIKV infections. Twenty-two samples exhibited Nab anti-CHIKV^+^RRV^+^, with the RRV/CHIKV Nab titre ≥ 4. These, along with one sample displaying single anti-RRV Nab, were reclassified as potential RRV infections (Table 2).

Sample No.4, 37, 38, and 40 were diagnosed as double RRV/CHIKV exposures, as they exhibited Nab anti-RRV^+^CHIKV^+^ without dominant Nab titres. Sample No.38 showed anti-RRV IgM^+^, suggesting a recent RRV infection. The recent infection status for sample No.4, 37, and 40 could not be determined as they either lacked IgM or displayed double IgM^+^.

Five samples (sample No.1, 2, 7, 9 and 16) exhibited triple Nab^+^. Among these samples, No.2, 7, 9, and 16 displayed the RRV/CHIKV Nab titre ≥ 4-fold. Therefore, these four samples, along with No.24 showing anti-RRV^+^BFV^+^ Nab, can be considered possible double RRV^+^/BFV^+^ infections (Table 2). The conclusions for sample No.1 were inconclusive, as the Nab titre for RRV/CHIKV/BFV = 640/160/40. The presence of CHIKV Nab positivity may be attributed to cross-reactivity from RRV, and the BFV titre may be due to cross-reactivity from CHIKV Nab. Given that these samples either lacked IgM or displayed triple IgM^+^ (No. 7), the virus responsible for the recent infection could not be determined.

Considering that only eight samples were diagnosed as potential CHIKV infection and four samples as possible RRV^+^/CHIKV^+^ co-infections, the rate of false positive CHIKV test results could be as high as 71.4% (30/42).

## 4. Discussion

Our study results indicated that there might be a potentially significant number of cases in which infection with RRV and BFV within this small cohort of patients were incorrectly diagnosed as CHIKV infection. This study underscores the issue of misdiagnosing RRV and BFV infection as CHIKV infection, particularly in settings where limited data were previously available in Australia.

Several factors could contribute to false positive CHIKV ELISA IgG/IgM results when using commercially available ELISA IgG/IgM kits. Firstly, the cross-reactivity among these alphaviruses may lead to false positive IgG/IgM results, as these viruses share conserved amino acid sequences. We found that amongst the fifteen single RRV-positive controls, six had anti-CHIKV IgG^+^, six had anti-CHIKV IgM^+^, one had anti-BFV IgG^+^ and one had anti-BFV IgM^+^. Additionally, two CHIKV IgG^+^ controls also had anti-RRV IgG^+^. This result corresponds with a previous report finding that CHIKV-infected human convalescent blood samples exhibited broad cross-reactivity against other alphaviruses within the Semliki Forest complex, including RRV and BFV [25]. Secondly, the varied sensitivity and specificity of commercial ELISA kits may also contribute to false positives. While Euroimmun claims that their IgG and IgM kits have good sensitivity and specificity values [26,27], their anti-CHIKV IgG instruction booklet indicates that up to 30% of RRV and 2.2% of BFV samples are positive for anti-CHIKV IgG. For the anti-CHIKV IgM kit, these numbers are 45% for RRV and 4.3% for BFV, respectively. The lower specificity (82%) of the Euroimmun CHIKV IgM kit has been reported [28], suggesting that false CHIKV positives could occur when using these kits to test the RRV/BFV-infected sera. Thirdly, the clinician’s interpretation of the laboratory results without caution could contribute to the issue. As most alphaviral infections are self-limiting and no specific anti-viral drug is available, clinicians may not conduct additional laboratory testing to exclude RRV and BFV infections, particularly if patients are no longer in the acute disease phase. While a four-fold rise in paired serology titres was traditionally used as a diagnostic criterion, such data are no longer routinely ordered by clinicians [16], potentially leading to deviations from the Australian health department CHIKV definition. Our results further suggest that these current commercially available CHIKV ELISA IgG/IgM kits cannot reliably differentiate the true virus infection in Australia, where the RRV/BFV virus is co-circulating. This emphasizes the need for improved diagnostic tools and greater awareness among clinicians so that alphviral infections can be accurately diagnosed and differentiated between.

The anti-viral Nab assay remains the gold standard test method for the confirmation of recent/past alphavirus infection, with high specificity and correlation with immune protection. However, these assays have limitations, including a slower onset of Nab generation, the need for live virus, differences in virus strains, the need for highly trained technicians, their labour intensity, longer turnaround times, and inability to differentiate between IgG and IgM antibodies. The lack of a national standard Nab procedure complicates the comparison of Nab results from different laboratories and the establishment of the threshold of protection. These challenges may explain why most public laboratories do not perform Nab assays. The Nab testing of our limited RRV, BFV, or CHIKV single-virus-infected sera suggests that this assay is not completely immune to cross-reactivity (Table 1) [23]. However, we can apply the traditional Nab ≥ 4-fold difference rule between the truly infected virus and the potential cross-reactive viruses to determine the single or multiple infections of these samples (Table 1 and Table 2).

The co-circulation and simultaneous co-infection of dengue, Chikungunya, and Zika viruses in patients with febrile syndrome have also been reported [29,30]. Double alphavirus infection with one alphavirus infection at an earlier time and another alphavirus infection later, or co-infection with two alphaviruses at the same time, is possible due to the sharing and overlapping of mosquito vectors and geographical locations, particularly in endemic areas of Australia. In these scenarios, double and triple-positive Nab results might not be solely caused by the cross-reactivity of the corresponding antibodies. Four samples were both Nab CHIKV^+^/RRV^+^, with no predominate virus identified, meeting the criteria of double RRV^+^/CHIKV^+^ infections. Five samples met the RRV^+^/BFV^+^ criteria and sample No.1 was diagnosed as possible CHIKV^+^/RRV^+^ or RRV^+^/BFV^+^. Since none of these simples had single IgM^+^, we were unable to determine if these nine patients were co-infected with multiple viruses simultaneously or infected with one virus first, followed by infection with another virus later. Notably, the exception was sample No.38, which had anti-RRV IgM^+^, indicating possible recent RRV infection. Interestingly, the mean age for double virus exposures was 60.5 (Table 1), significantly higher than the mean age of the whole cohort, which was 46.5. Generally, older individuals have a higher chance of exposure to multiple viruses in their lifetime.

The positive neutralizing results for ELISA^-^negative or borderline samples could be attributed to antigenic differences between the circulating virus linages and the virus antigen used for ELSIA and Nab assays [31]. Additionally, the sensitivity and specificity of ELISA kits may also contribute to this phenomenon [32,33].

BFV, CHIKV and RRV are notifiable diseases in Australia, each with clear case definitions provided by the Australian Health Department [18,19,34]. Therefore, single CHIKV IgM/IgG ELISA-positive results need to be interpreted with caution. To exclude RRV and BFV infections, it is essential to consider travel history, clinical symptoms and conduct further laboratory tests [16,35,36,37]. Unfortunately, only three patients in our study had a clear overseas travel history and no other patient travel histories were available. If these patients were reported as notifiable CHIKV cases, reference laboratories should confirm these patient samples with Nab assays against all three viruses and complete their travel history. This step is crucial to exclude the possibility that they were infected in Australia. Since CHIKV is not endemic in Australia, a locally acquired case of this virus would warrant several health measures and vector control. Australia faces an increased risk of CHIKV becoming endemic due to overseas travel, rising foreign-acquired cases, and the presence of *Ae. aegypti* mosquitoes in northern Queensland.

To address the challenges associated with the low specificity of ELISA IgG/IgM, the increase in labour, and the high cost of Nab assays, there is an urgent need for new serologic methods that can differentiate among these alphaviruses [37,38,39]. The use of commercial and in-house peptide ELISAs to detect antibodies against specific epitopes of structure proteins [40,41,42,43], along with the utilization of virus specific monoclonal antibodies for competitive ELISAs, may offer solutions that resolve the cross-reactivity among alphaviruses. However, it is essential that these new methods are extensively validated before being applied in routine diagnostics. Additionally, employing parallel quantitative ELISAs for BFV, CHIKV and RRV, rather than the current single-well semi-quantitative ELISA, may provide a convenient and robust solution that ensures specificity and differentiation in diagnosis [44].

This preliminary finding necessitates further investigations, given the small sample size and the fact that all antibody positives resulted from a single IgG/IgM and Nab test as we could not repeat the assays due to limitations regarding the sample volume. However, the results presented here offer insight into the potential misdiagnosis of RRV and BFV as CHIKV infections in Australia. The authors plan to expand their research in collaboration with public laboratories, to investigate the further misdiagnosis of infection with these three-alphaviruses in Australia.

## Figures and Tables

**Table 1:** The anti-alphavirus antibody characteristics of our single RRV, BFV, or CHIKV virus-infected personnel and negative controls.

Serum No.	Anti-BFV			Anti-RRV			Anti-CHIKV		
	IgG	IgM	Nab	IgG	IgM	Nab	IgG	IgM	Nab
RRV-Positive 1	-	-	0	+	+	320	+	+	0
RRV-Positive 2	-	±	0	+	-	320	+	-	0
RRV-Positive 3	-	±	0	+	-	320	-	-	20
RRV-Positive 4	-	+	0	+	+	320	+	-	0
RRV-Positive 5	-	-	0	+	+	160	-	+	0
RRV-Positive 6	-	-	0	+	-	640	+	-	0
RRV-Positive 7	-	-	0	+	-	160	+	±	10
RRV-Positive 8	-	-	0	-	+	10	-	-	10
RRV-Positive 9	-	-	0	+	+	320	-	+	10
RRV-Positive 10	+	-	0	+	+	320	-	+	10
RRV-Positive 11	-	-	0	+	+	20	-	+	10
RRV-Positive 12	-	-	0	+	+	80	-	±	0
RRV-Positive 13	-	-	0	+	+	10	-	±	10
RRV-Positive 14	-	-	0	+	+	640	+	+	10
Panbio RRV Kit IgG positive control	-	-	0	+	-	320	+	-	10
BFV-positive 1	+	-	160	-	-	0	-	-	0
BFV-positive 2	+	+	80	±	+	0	±	-	0
BFV-positive 3	+	-	40	-	-	0	±	-	0
BFV-positive 4	+	-	160	-	-	0	-	-	0
BFV-positive 5	+	+	320	-	+	0	-	-	0
BFV-positive 6	+	±	160	-	-	0	-	-	10
CHIKV-positive 1	-	-	0	+	-	10	+	-	640
CHIKV-positive 2	±	-	0	+	-	20	+	-	640
Negative 1	-	-	0	-	-	0	-	-	0
Negative 2	-	-	0	-	-	0	-	-	0
Negative 3	-	-	0	-	-	0	-	-	0
Negative 4	-	-	0	-	-	0	-	-	0
Negative 5	-	-	0	-	-	0	-	-	0

+ = positive; - = negative; ± = equivalent.

**Table 2:** Anti-alphavirus antibody characteristics and the possible virus infection diagnosis for these 42 patients.

Patient ID	Age *	Anti-BFV			Anti-CHIKV			Anti-RRV			Diagnosis
		IgG	IgM	NAb	IgG	IgM	NAb	IgG	IgM	NAb	
**No. 1**	**56**	**+**	**−**	**40**	**−**	**−**	**160**	**+**	**−**	**640**	**RRV + CHIKV (CHIKV/BFV = 4)** **Or RRV + BFV (RRV/CHIKV = 4)**
**No. 2**	**81**	**+**	**±**	**160**	**+**	**−**	**40**	**+**	**−**	**320**	**RRV + BFV** (RRV/CHIKV = 8, plus BFV^+^)
No. 3	33	−	−	0	+	+	160	+	+	640	RRV (RRV/CHIKV = 4)
**No. 4**	**57**	**−**	**−**	**0**	**+**	**−**	**320**	**+**	**−**	**320**	**RRV + CHIKV (RRV/CHIKV = 1)**
No. 5	26	±	−	0	+	±	320	+	−	0	CHIKV (single CHIKV Nab^+^)
No. 6	44	−	−	0	+	+	40	+	±	320	RRV (RRV/CHIKV = 8)
**No. 7**	**65**	**+**	**+**	**640**	**+**	**+**	**40**	**+**	**+**	**160**	**RRV + BFV** (RRV/CHIKV = 4, plus BFV^+^)
No. 8	39	−	−	0	+	±	20	+	−	80	RRV (RRV/CHIKV = 4)
**No. 9**	**71**	**+**	**−**	**80**	**+**	**−**	**20**	**+**	**−**	**80**	**RRV + BFV** (RRV/CHIKV = 4, plus BFV^+^)
No. 10	56	−	−	0	±	−	20	+	−	80	RRV (RRV/CHIKV = 4)
No. 11	72	−	−	0	+	+	80	+	+	320	RRV (RRV/CHIKV = 4)
No. 12	49	−	−	0	+	±	80	+	+	320	RRV (RRV/CHIKV = 4)
No. 13	44	+	−	0	+	−	640	+	−	20	CHIKV (CHIKV/RRV = 32)
No. 14	66	−	−	0	+	−	10	+	−	320	RRV (RRV/CHIKV = 32)
No. 15	12	−	−	0	+	±	10	+	−	320	RRV (RRV/CHIKV = 32)
**No. 16**	**79**	**+**	**−**	**160**	**+**	**−**	**20**	**+**	**−**	**160**	**RRV + BFV** (RRV/CHIKV = 8, plus BFV^+^)
No. 17	36	−	−	0	+	−	10	+	−	320	RRV (RRV/CHIKV = 32)
No. 18	38	−	−	0	+	+	10	+	+	160	RRV (RRV/CHIKV = 16)
No. 19	77	−	−	0	+	−	40	+	+	640	RRV (RRV/CHIKV = 16)
No. 20	71	−	−	0	+	−	40	+	−	640	RRV (RRV/CHIKV = 16)
No. 21	40	−	−	0	+	+	20	+	−	320	RRV (RRV/CHIKV = 16)
No. 22	32	−	−	0	+	−	10	+	−	160	RRV (RRV/CHIKV = 16)
No. 23	57	+	−	20	±	−	0	±	−	0	BFV (single BFV^+^)
**No. 24**	**62**	**−**	**−**	**20**	**+**	**−**	**0**	**+**	**−**	**160**	**RRV + BFV (RRV^+^BFV^+^)**
No. 25	39	−	−	0	+	±	20	+	−	0	CHIKV (single CHIKV^+^)
No. 26	34	+	−	0	+	−	160	±	−	0	CHIKV (single CHIKV^+^)
No. 27	49	−	−	0	+	+	20	+	+	160	RRV (RRV/CHIKV = 8)
No. 28	40	−	−	0	+	±	40	+	+	640	RRV (RRV/CHIKV = 16)
No. 29	55	−	−	0	+	±	10	+	−	160	RRV (RRV/CHIKV = 16)
No. 30	31	−	−	0	+	−	10	+	±	160	RRV (RRV/CHIKV = 16)
No. 31	53	−	−	0	+	+	0	+	±	320	RRV (single RRV^+^)
No. 32	48	−	−	0	+	±	40	−	−	0	CHIKV (single CHIKV^+^)
No. 33	61	−	−	0	+	−	20	−	−	0	CHIKV (single CHIKV^+^)
No. 34	39	−	−	0	+	−	20	+	−	160	RRV (RRV/CHIKV = 8)
No. 35	38	−	−	0	+	−	20	+	+	160	RRV (RRV/CHIKV = 8)
No. 36	66		−	0	+	−	10	+	+	80	RRV (RRV/CHIKV = 8)
**No. 37**	**19**	**−**	**−**	**0**	**+**	**+**	**80**	**+**	**+**	**160**	**RRV + CHIKV (RRV/CHIKV = 2)**
**No. 38**	**61**	**−**	**−**	**0**	**+**	**±**	**20**	**+**	**+**	**40**	**RRV + CHIKV (RRV/CHIKV = 2)**
No. 39	49	±	−	0	+	−	640	+	−	10	CHIKV (CHIKV/RRV = 64)
**No. 40**	**41**	**−**	**−**	**0**	**+**	**−**	**40**	**+**	**−**	**80**	**RRV + CHIKV (RRV/CHIKV = 2)**
No. 41	68	−	−	0	+	−	40	+	−	160	RRV (RRV/CHIKV = 4)
No. 42	62	−	−	0	−	±	20	−	−	0	CHIKV (Single CHIKV^+^)

+ = positive; − = negative; ± = equivalent. * The double infections are highlighted with bold font.

**Table 3:** Summary of the anti-Barmah Forest virus, anti-Chikungunya virus and anti-Ross River virus antibody characteristics of this study.

	Anti-BFV	Anti-CHIKV	Anti-RRV
IgG^+^	19%(8/42)	90.5%(38/42)	88.1%(37/42) *
IgG^−^	76.2%(32/42)	4.8%(2/42)	7.1%(3/42)
IgG^±^	4.8%(2/42)	4.8%(2/42)	4.8%(2/42)
IgM^+^	2.4%(1/42)	21.4%(9/42)	28.6%(12/42) *
IgM^−^	95.2%(40/42)	54.7%(23/42)	64.3%(27/42)
IgM^±^	2.4%(1/42)	23.8%(10/42)	7.1%(3/42)
IgG^+^+IgM^+^	2.4%(1/42)	21.4%(9/42)	28.6%(12/42) *
Nab^+^	16.7%(7/42)	93.3%(39/42)	83.3%(35/42)
Nab^−^	83.3%(35/42)	7.1%(3/42)	7%(7/42)
*ELISA^+^Nab^+^	4.8%(6/42)	90.5%(38/42)	83.3%(35/42) *
*ELISA^+^Nab^−^	2.4%(1/42)	4.8%(2/42)	4.8%(2/42)
#ELISA^−^Nab^+^	0%(0/42)	4.8%(2/42)	0%(0/42)
#ELISA^−^Nab^−^	83.3%(35/42)	0%(0/42)	11.9%(5/42)

* No significant statistical difference between RRV and CHIKV positivity among these patients.

**Table 4:** Multiple antibody positivity among these samples.

	BFV^+^/CHIKV^+^	BFV^+^/RRV^+^	CHIKV^+^/RRV^+^	BFV^+^/CHIKV^+^/RRV^+^
IgG^+^	2.4%(1/42)	2.4%(1/42)	71.4%(30/42)	11.9%(5/42)
IgM^+^	0%(0/42)	0%(0/42)	11.9%(5/42)	2.4%(1/42)
Nab^+^	0%(0/42)	0%(0/42)	69.1%(29/42)	11.9%(5/42)

## Data Availability

The data is included in the manuscript.
